# Supplementary material for: Altered microRNA Transcriptome in Cultured Human Liver Cells upon Infection with Ebola Virus
Source: Int J Mol Sci. 2021 Apr 6;22(7):3792. doi: 10.3390/ijms22073792 (PMC8038836; doi:10.3390/ijms22073792)
Supplement: Supplementary file 1 [file ijms-22-03792-s001.zip › Supplementary_File/C_ GO_Analysis_Results/16-30nt_go_Reston-96h-Huh7_vs_Control-96h-Huh7_down.mature_mirna_targets/GeneOntology_EnrichmentScore.pdf]

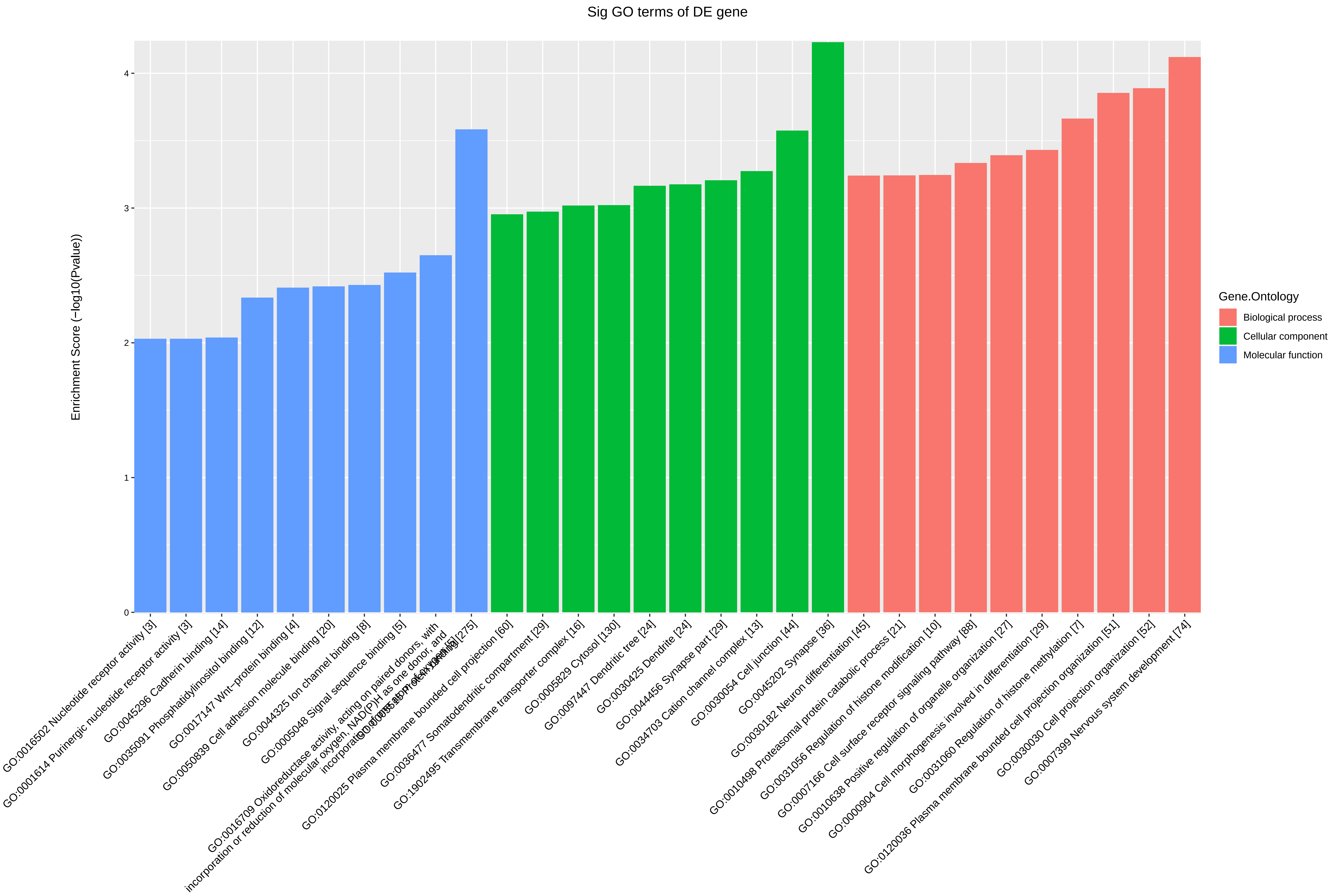

Sig GO terms of DE gene
Enrichment Score (−log10(Pvalue))
0
1
2
3
4
GO:0016502 Nucleotide receptor activity [3]
GO:0001614 Purinergic nucleotide receptor activity [3]
GO:0045296 Cadherin binding [14]
GO:0035091 Phosphatidylinositol binding [12]
GO:0017147 Wnt−protein binding [4]
GO:0050839 Cell adhesion molecule binding [20]
GO:0044325 Ion channel binding [8]
GO:0005048 Signal sequence binding [5]
GO:0016709 Oxidoreductase activity, acting on paired donors, with incorporation or reduction of molecular oxygen, NAD(P)H as one donor, and incorporation of one atom of oxygen [5]
GO:0005515 Protein binding [275]
GO:0120025 Plasma membrane bounded cell projection [60]
GO:0036477 Somatodendritic compartment [29]
GO:1902495 Transmembrane transporter complex [16]
GO:0005829 Cytosol [130]
GO:0097447 Dendritic tree [24]
GO:0030425 Dendrite [24]
GO:0044456 Synapse part [29]
GO:0034703 Cation channel complex [13]
GO:0030054 Cell junction [44]
GO:0045202 Synapse [36]
GO:0030182 Neuron differentiation [45]
GO:0010498 Proteasomal protein catabolic process [21]
GO:0031056 Regulation of histone modification [10]
GO:0007166 Cell surface receptor signaling pathway [88]
GO:0010638 Positive regulation of organelle organization [27]
GO:0000904 Cell morphogenesis involved in differentiation [29]
GO:0031060 Regulation of histone methylation [7]
GO:0120036 Plasma membrane bounded cell projection organization [51]
GO:0030030 Cell projection organization [52]
GO:0007399 Nervous system development [74]
Gene.Ontology
Biological process
Cellular component
Molecular function
